# *In Vitro* Screening of 10 Edible Thai Plants for Potential Antifungal Properties

**DOI:** 10.1155/2014/138587

**Published:** 2014-01-02

**Authors:** Supattra Suwanmanee, Thitinan Kitisin, Natthanej Luplertlop

**Affiliations:** ^1^Department of Tropical Pathology, Faculty of Tropical Medicine, Mahidol University, Bangkok 10400, Thailand; ^2^Department of Anatomy, Faculty of Science, Mahidol University, Bangkok 10400, Thailand; ^3^Department of Microbiology and Immunology, Faculty of Tropical Medicine, Mahidol University, Bangkok 10400, Thailand

## Abstract

Growing rates of fungal infections and increasing resistance against standard antifungal drugs can cause serious health problems. There is, therefore, increasing interest in the potential use of medicinal plants as novel antifungal agents. This study investigates the antifungal properties of crude plant extracts from ten medicinal plant species. Crude samples were extracted using the hot water extraction process. The minimum inhibitory concentrations (MIC) and diameter zone of inhibition were determined in each extract against ten fungal strains, and fluconazole was used as a positive control. The cytotoxicity of crude extracts on *in vitro* human skin fibroblast (HSF) cell models was determined by MTT assay. Of the ten crude extracts, *Psidium guajava* L. exhibited the highest antifungal activity, diameter zone of inhibition, and percentage HSF cell viability. Although all extracts exhibited antifungal activity, *Psidium guajava L*. had the greatest potential for developing antifungal treatments.

## 1. Introduction

Despite the intensive prophylactic use of antifungal drugs, the incidence of fungal infections has increased due to growing resistance [1, 2]. Therefore, the possibility of finding novel antifungal agents from crude plant extracts has been explored. Many edible plants are known to have beneficial and medicinal properties to humans, and plant extracts have been used as a source of alternative medication for their antioxidative, antifungal, and anticancer properties [3].

Ten plants commonly used in Thai traditional medicine were selected for this study: *Garcinia mangostana* L., *Piper betel* L., *Camellia sinensis*, *Morus alba* Linn., *Aegle marmelos* L., *Garcinia atroviridis* Griff., *Andrographis paniculata (Burm.f.)* Wall.ex Nees, *Schefflera leucantha* R. Vig., *Carthamus tinctorius* L., and *Psidium guajava* L. The objective of this study was to evaluate the *in vitro* antifungal activities of crude extracts of these edible plant species in a systematic and consistent manner against clinical pathogens. The results indicate that there are potential benefits of using crude plant extracts for the treatment of fungal infections.

## 2. Materials and Methods

### 2.1. Preparation of Plant Sample Extracts

The ten plant species used in this study can be divided into four categories according to their active ingredients. The first category is condensed tannins, which is obtained from the peel of *Garcinia mangostana* L. (mangosteen). The second is hydrolysable tannins, obtained from the leaf of *Piper betel* L. (betel vine) and *Camellia sinensis* (green tea). The third category is flavonols, obtained from the leaf of *Morus alba* Linn. (mulberry). The fourth category is phenols, obtained from the fruit of *Aegle marmelos* L. (bael) and *Garcinia atroviridis* Griff (Malabar tamarind), from the leaf of *Andrographis paniculata* (Burm.f.) Wall.ex Nees (create), *Schefflera leucantha* R. Vig. (edible-stemmed vine), and *Psidium guajava* L. (guava), and from the flower of *Carthamus tinctorius* L. (safflower) (Table 1). All plant samples were provided by the Faculty of Cosmetic Science, Mae Fah Luang University, and were collected from northern Thailand, Chiang Rai province. The crude plant extraction process was performed according to AOAC procedures, with some modifications [4]. 100 g of each plant sample was dried, powdered, macerated, and extracted by hot water extraction process. Subsequently, all crude extracts underwent sterile filtering by using a 0.22 *μ*m sterile filter. All sterile filtered crude extracts were stored at −20°C. The crude extracts were later thawed and serial dilutions were prepared by using Sabouraud Dextrose Broth (SDB) media.

### 2.2. Fungal Strains and Growth Conditions

Fungal isolates were subcultured and prepared for assessment of the antifungal properties of the plant extracts. All fungal strains used in this study were provided by the Department of Microbiology and Immunology, Faculty of Tropical Medicine, Mahidol University, Thailand. The fungi can be divided into 3 groups. The first group is yeasts, which include *Candida albicans* and *Saccharomyces cerevisiae*. The second is dermatophytes, which include *Trichophyton mentagrophytes*, *Trichophyton rubrum*, *Trichophyton tonsurans*, *Epidermophyton floccosum*, *Microsporum canis*, and *Microsporum gypseum*. The third group is filamentous fungi, which include *Aspergillus niger* and *Penicillium* spp. All fungi were cultured in Sabouraud agar slants or Sabouraud Dextrose Broth (SDB). The clinical fungal strains used in this study are listed in Table 2. The yeasts were each diluted in SDB broth to a concentration of 10^6^ CFU/mL. This was further diluted with RPMI to 2 × 10^5^ CFU/mL, and 100 *μ*L was then added to each of the serial dilutions of the plant extracts. 100 *μ*L of each dilution was then added to the serial dilutions of the plant extracts.

### 2.3. Antifungal Activity

The screening of plant extracts for antifungal activity was investigated using a modified broth microdilution method. The plant extract/fungal isolate microtiter trays were stored at 35°C and assessed for growth after 48 h. The antifungal agents were prepared for minimum inhibitory concentration (MIC), according to Clinical and Laboratory Standards Institute (CLSI-formerly NCCLS) [16, 17] with RPMI-MOPS (RPMI 1640 medium containing l-glutamine, without sodium bicarbonate (Sigma-Aldrich Co., St. Louis, USA) buffered to pH 7.0 with 0.165 mol/L MOPS buffer-Sigma). The SDB media were used as negative control, and fluconazole (1 mg/mL) was used as positive control. MIC was represented as the lowest concentration of compounds at which the microorganism tested did not demonstrate visible growth. The experiments were carried out in triplicate. Inhibition (%) was calculated as follows:

(1) $$\begin{array}{c} \text{Inhibition}\;\left(\%\right)=\left[\frac{\left(\text{DC}-\text{DE}\right)}{\text{DC}}\right]\times 100, \end{array}$$

where DE is the diameter of growth zone in experimental disc (cm) and DC is the diameter of the growth zone in the control disc (cm).

### 2.4. Human Skin Fibroblast (HSF) Cell Preparation

Human skin fibroblast (HSF) cells were maintained in Dulbecco's modified minimum essential medium (DMEM, Gibco, USA), supplemented with 10% heat-inactivated (56°C for 30 min) fetal bovine serum (FBS, Hycone, USA), 1% L-glutamine, and 1% antibiotics (200 U/mL penicillin and 100 *μ*g/mL of streptomycin, Gibco, USA) at 37°C with 5% concentration of CO_2_ incubator. The cells were detached from the surface using 0.25% trypsin/EDTA (PAA Laboratories GmbH, Austria).

### 2.5. Effect of Plant Extracts on Cell Viability (MTT) on Human Skin Fibroblast (HSF) Cells

The colorimetric MTT assay used was similar to that originally described by Mosmann [18]. The assay was based on the ability of mitochondria to reduce MTT, a yellow tetrazolium dye, to MTT formazan, a blue mitochondrial byproduct. The reduction is mediated by mitochondrial dehydrogenases that are present in living but not dead cells.

HSF cells were cultured in 96-well cell-culture plates with a cell concentration of 1.0 × 10^5^ cells/mL. 200 *μ*L of the diluted cell suspension transferred to each well. The cells were treated with 200 *μ*L of crude plant extract samples. Fluconazole was used as positive control and SDB as negative control at 37°C for 24 hours, in triplicate, followed by cell washing. 20 *μ*L of MTT (0.1 mg/mL) solution was added to each well. After 4 hours, the supernatant was aspirated and 150 *μ*L of DMSO was added to each well; then, the cells were incubated at 37°C for 10 minutes. The absorbance at 570 nm was then read for each well to determine cell viability. The lethal dose of cytotoxic concentration from crude plant extract samples was expressed as 50% cellular mitochondrial activity, LD_50_ (mg/mL) and was obtained by interpolation from linear regression analysis.

### 2.6. Statistical Analysis

All assays were conducted in triplicate and unpaired *t*-tests of independent experiments were performed by statistical analysis using GraphPad Prism 6 program version 6.02 (Trial) (Statcon). Results were considered significant at *P* value ≤ 0.05.

## 3. Results and Discussion

The results of our antifungal activity analysis revealed that the MIC values from crude plant extracts varied from 2.67 mg/mL to 128 mg/mL. Different fungal strains exhibited different levels of sensitivity to each crude plant extract.  Table 3 indicates that crude extracts from *Psidium guajava* L. exhibited the highest antifungal activity, diameter zone of inhibition, LD_50_, and percent HSF cell viability, while *Aegle marmelos* L. exhibited the lowest activity compared to the other plants (*Psidium guajava* L. > *Piper betel* L. > *Schefflera leucantha* R. Vig > *Andrographis paniculata* (Burm.f.) Wall.ex Nees > *Garcinia atroviridis* Griff > *Morus alba* Linn. > *Garcinia mangostana* L. > *Carthamus tinctorius* L. > *Camellia sinensis* > *Aegle marmelos* L.).

*Psidium guajava* L. crude extracts were extracted from the plant's leaves. Previous studies have found that *Psidium guajava* L. leaves have high levels of essential oils, primarily *β*-pinene, *α*-pinene, limonene, menthol, terpenyl acetate, isopropyl alcohol, longicyclene, caryophyllene, *β*-bisabolene, cineol, caryophyllene oxide, *β*-copanene, farnesene, humulene, selinene, cardinene, and curcumene [13, 14]. High quantities of flavonoids and triterpenoids were also found [15]. In addition to these active compounds found in *Psidium guajava* L., this study found that *Psidium guajava L.* crude extracts exhibited the highest antifungal activity of the plants tested (Table 3), supporting previous studies of the plant's beneficial properties [19–22].

*Piper betel* L. crude extracts, also obtained from the leaf, showed the second highest antifungal activity compared with the other plants tested (Table 3). A preliminary study has reported that *Piper betel* L. leaf extract contains a large number of bioactive molecules, including polyphenols, alkaloids, steroids, saponins, and hydrolysable tannins [6]. The crude extracts containing these active ingredients exhibited fungal cytotoxicity against all fungal strains tested, supporting previous investigations [23].

*Schefflera leucantha* R. Vig crude (leaf) extracts showed the third highest antifungal activity compared with the other plants (Table 3). It has been reported that the active compound found in the crude extract is a mixture of saponins [11].

*Andrographis paniculata* (Burm.f.) Wall.ex Nees (leaf) extracts contain phytochemicals such as DL-limonene and euasarone [10].

Atroviridin, garcinia acid, and *γ*-lactone were major active compounds found in *Garcinia atroviridis* Griff (fruit) extracts [5].

Papyriflavonol A, kuraridin, sophoraflavanone D and sophoraisoflavanone A were previously identified as antifungal compounds found in *Morus alba* Linn (leaf) extracts [8].

*α*-mangostin was a major antifungal compound found in *Garcinia mangostana* L. (peel) extracts [24].

Coumaroylspermidine was found in *Carthamus tinctorius* L. (flower) extracts and reportedly has antifungal activity [12].

The active catechin compounds were the most beneficial effects from *Camellia sinensis* (leaf) extracts [7], which exhibited antifungal and anti bacterial activities [25].

Aegelenine and aegeline, phlobatannins, flavan-3-oil, leucoanthocyanins anthocyanins, flavonoid glycosides, skimmianine, *β*-sitosterol, rutin, and marmesinin have been found in leaf extracts of *Aegle marmelos* L., which exhibited antifungal activity [9].

Interestingly, our study shows that the highest antifungal activity derived from crude extracts containing active ingredients found in the leaf (such as *Psidium guajava* L. and *Piper betel* L.). However, one should note that the phytochemical properties from extracts in this study may vary depending on ecological and geographical conditions, age of plant, and time of harvesting. The exact level of active compound concentration in each extract also requires further study, as this information may provide valuable data for large-scale production of crude plant extracts with antifungal properties.

The high function of phenols in *Psidium guajava* L. crude extract exhibited an antioxidative property, which was reflected in high LD_50_ (3.50 mg/mL) compared with other crude plant extracts (Table 3). These data indicate that a high LD_50_ concentration value results in strong antifungal properties, and as determined by HSF cells *in vitro*, is also safe for application as a topical treatment. *Psidium guajava* L. crude extract, previously reported for antioxidative activity, [13–15] showed high % HSF cell viability (93%) (Table 3) compared with other plants and fluconazole (antifungal drug; LD_50_, 1.00 mg/mL and cell viability, 86%). This suggests that phenols found in crude plant extracts, particularly in *Psidium guajava* L., not only exhibit antifungal activity but also prevent cellular damage and oxidative injuries arising from free radicals or reactive oxygen species (ROS) [26]. This may be a promising result leading to safer antifungal agents for use on human skin.

Our findings show that all the crude plant extracts studied were effective antifungal agents. However, the phenol-based group exhibited the highest activity and may also help prevent cellular damage from oxidative stress through its antioxidative property, represented by high % cell viability.

## 4. Conclusion

Our study investigated the role of ten selected crude plant extracts and their antifungal activity. Among them, the phenol crude extract group exhibited outstanding results (particularly *Psidium guajava* L.). Our findings suggest an alternative way to utilize crude plant extracts as their antifungal activity and antioxidative properties may alleviate these pathogenic infections. However, further studies are needed, particularly on the properties of crude plant extracts on immunomodulation against fungi, the reduction of fungal infection-induced inflammatory cytokine after crude plant extract treatment both *in vitro* and *in vivo*, and the formulation of cosmetic products using active ingredient from plant extracts.

## Figures and Tables

**Table 1:** List of plant species and parts of the plant used to determine antifungal activity as crude plant extracts obtained by hot water extraction process.

Active ingredient	Group	Plant species with ref.	General name	Part used
Tannins	Condensed tannins	*Garcinia mangostana *L. [5]	Mangosteen	Peel
	Hydrolysable tannins	*Piper betel *L. [6]	Betel vine	Leaf
		*Camellia sinensis* [7]	Green tea	Leaf

Flavonoids	Flavonols	*Morus alba *Linn. [8]	Mulberry	Leaf

Phenol	Phenol	*Aegle marmelos *L. [9]	Bael	Fruit
		*Garcinia atroviridis *Griff. [5]	Malabar tamarind	Fruit
		*Andrographis paniculata *(Burm.f.) Wall. ex Nees [10]	Create	Leaf
		*Schefflera leucantha *R. Vig [11]	Edible-stemmed Vine	Leaf
		*Carthamus tinctorius *L. [12]	Safflower	Flower
		*Psidium guajava *L. [13–15]	Guava	Leaf

**Table 2:** Clinical human pathogenic fungal strains used in this study.

Type	Name
Yeast	*Candida albicans *
	*Saccharomyces cerevisiae *

Dermatophytes	*Trichophyton mentagrophytes *
	*Trichophyton rubrum *
	*Trichophyton tonsurans *
	*Epidermophyton floccosum *
	*Microsporum canis *
	*Microsporum gypseum *

Filamentous fungi	*Aspergillus niger *
	*Penicillium *spp.

**Table 3:** Minimum inhibitory concentration (MIC; mg/mL), inhibition zone diameter (mm) at 48 hr after treatment, % inhibition zone, and cytotoxicity activity (LD_50_ and % cell viability on human skin fibroblast cells) on crude plant extracts.

Plant names (part used)	Antifungal activity	Fungal strains										HSF cells	
		*C*. *al *	*S*. *ce *	*T*. *men *	*T*. *rub *	*T*. *tons *	*E*. *flo *	*M*. *can *	*M*. *gyp *	*A*. *ni *	*P*. spp	LD_50_ (mg/mL)	Cell viability (%)
*Garcinia mangostana L.* (peel)	MIC	106.67	85.33	128	>128	128	>128	>128	>128	>128	>128	1.25	74 ± 3.5
	Inhibition zone (D)	5 ± 0.75	5 ± 0.44	2 ± 0.48	1 ± 0.08	2 ± 0.01	1 ± 0.07	1 ± 0.1	1 ± 0.74	1 ± 0.04	1 ± 0.95		
	Inhibition (%)	11.90 ± 0.75	12.82 ± 0.44	5 ± 0.48	3.13 ± 0.08	7.14 ± 0.01	3.7 ± 0.07	3.57 ± 0.1	4.35 ± 0.74	2.22 ± 0.04	2.5 ± 0.95		

*Piper betel *L. (leaf)	MIC	16	42.67	8	4	5.3	6.6	10.67	10.67	21.33	26.67	3.10	93 ± 0.95
	Inhibition zone (D)	13 ± 1.96	7 ± 0.76	12 ± 1.33	12 ± 1.02	17 ± 0.83	12 ± 0.95	11 ± 0.32	11 ± 2.38	1 ± 1.36	9 ± 1.33		
	Inhibition (%)	30.95 ± 1.96	17.95 ± 0.76	30 ± 1.33	37.5 ± 1.02	50 ± 0.83	44.44 ± 0.95	39.29 ± 0.32	47.83 ± 2.38	2.22 ± 1.36	22.5 ± 1.33		

*Camellia sinensis* (leaf)	MIC	26.67	26.67	18.67	42.67	16	13.33	21.33	18.67	53.33	42.67	2.70	89 ± 3.2
	Inhibition zone (D)	2 ± 0.86	2 ± 0.12	5 ± 2.75	1 ± 0.09	2 ± 0.09	2 ± 0.01	2 ± 0.72	2 ± 0.03	1 ± 0.93	1 ± 0.08		
	Inhibition (%)	4.76 ± 0.86	5.13 ± 0.12	12.5 ± 2.75	3.13 ± 0.09	7.14 ± 0.09	7.41 ± 0.01	7.14 ± 0.72	8.69 ± 0.03	2.22 ± 0.93	2.5 ± 0.08		

*Morus alba *Linn. (leaf)	MIC	32	64	26.67	85.33	106.67	85.33	53.33	42.67	106.67	128	1.85	77 ± 2.8
	Inhibition zone (D)	3 ± 0.92	2 ± 0.59	3 ± 0.36	1 ± 0.1	1 ± 0.15	2 ± 0.85	2 ± 0.18	2 ± 0.1	1 ± 0.76	1 ± 0.91		
	Inhibition (%)	7.14 ± 0.92	5.13 ± 0.59	7.5 ± 0.36	3.13 ± 0.1	3.57 ± 0.15	7.41 ± 0.85	7.14 ± 0.18	8.69 ± 0.1	2.22 ± 0.76	2.5 ± 0.91		

*Aegle marmelos *L. (fruit)	MIC	53.33	16	37.33	37.33	26.67	21.33	37.33	26.67	53.33	64	2.55	85 ± 2.1
	Inhibition zone (D)	1 ± 0.74	4 ± 0.87	1 ± 0.62	1 ± 0.38	1 ± 0.33	1 ± 0.03	1 ± 0.54	1 ± 0.07	1 ± 0.48	1 ± 0.75		
	Inhibition (%)	2.38 ± 0.74	10.26 ± 0.87	2.5 ± 0.62	3.13 ± 0.38	3.57 ± 0.33	3.7 ± 0.03	3.57 ± 0.54	4.35 ± 0.07	2.22 ± 0.48	2.5 ± 0.75		

*Garcinia atroviridis *Griff (fruit)	MIC	10.67	16	3.33	10.67	2.67	3.33	4	4	13.33	13.33	1.30	71 ± 0.9
	Inhibition zone (D)	7 ± 0.88	3 ± 0.91	11 ± 1.34	7 ± 1.33	12 ± 0.99	9 ± 1.11	9 ± 1.66	8 ± 1.14	3 ± 1.14	4 ± 1.13		
	Inhibition (%)	16.67 ± 0.88	7.69 ± 0.91	27.5 ± 1.34	21.88 ± 1.33	42.86 ± 0.99	33.33 ± 1.11	32.14 ± 1.66	34.78 ± 1.14	6.67 ± 1.14	10 ± 1.13		

*Andrographis paniculata *(Burm.f.) Wall.ex Nees (leaf)	MIC	42.67	21.33	5.33	6.67	8	10.67	13.33	13.33	37.33	26.67	2.80	90 ± 2.4
	Inhibition zone (D)	2 ± 1.12	4 ± 0.33	9 ± 1.08	8 ± 0.85	9 ± 1.3	8 ± 0.95	8 ± 0.98	8 ± 1.02	3 ± 0.99	3 ± 0.86		
	Inhibition (%)	4.76 ± 1.12	10.26 ± 0.33	22.5 ± 1.08	25 ± 0.85	32.14 ± 1.3	29.63 ± 0.95	28.57 ± 0.98	34.78 ± 1.02	6.67 ± 0.99	7.5 ± 0.86		

*Schefflera leucantha *R.Vig (leaf)	MIC	9.33	16	9.33	9.33	5.33	8	12	13.33	21.33	26.67	2.90	91 ± 1.9
	Inhibition zone (D)	8 ± 1.10	5 ± 0.14	7 ± 0.92	6 ± 1.44	7 ± 0.65	9 ± 1.33	6 ± 1.21	6 ± 1.95	5 ± 1.13	4 ± 1.35		
	Inhibition (%)	19.05 ± 1.10	12.82 ± 0.14	17.5 ± 0.92	18.75 ± 1.44	25 ± 0.65	33.33 ± 1.33	21.43 ± 1.21	26.09 ± 1.95	11.11 ± 1.13	10 ± 1.35		

*Carthamus tinctorius *L. (flower)	MIC	37.33	53.33	53.33	48	48	53.33	26.67	53.33	64	26.67	2.00	79 ± 1.1
	Inhibition zone (D)	3 ± 0.34	3 ± 0.55	2 ± 0.35	2 ± 0.09	3 ± 0.48	2 ± 1.04	2 ± 0.8	3 ± 0.02	2 ± 0.75	3 ± 0.14		
	Inhibition (%)	7.14 ± 0.34	7.69 ± 0.55	5 ± 0.35	6.25 ± 0.09	10.72 ± 0.48	11.11 ± 1.04	7.14 ± 0.8	13.04 ± 0.02	4.44 ± 0.75	7.5 ± 0.14		

*Psidium guajava *L. (leaf)	MIC	6.67	16	3.33	2.67	2.67	4	5.33	3.33	13.33	6.67	3.50	93 ± 1.1
	Inhibition zone (D)	11 ± 1.83	7 ± 1.99	14 ± 1.21	13 ± 0.82	15 ± 0.77	13 ± 1.03	13 ± 1.15	12 ± 1.3	9 ± 1.27	11 ± 0.9		
	Inhibition (%)	26.19 ± 1.83	17.95 ± 1.99	35 ± 1.21	40.63 ± 0.82	53.57 ± 0.77	48.15 ± 1.03	46.43 ± 1.15	52.17 ± 1.3	20 ± 1.27	27.5 ± 0.9		

SDB	MIC/MFC	NT	NT	NT	NT	NT	NT	NT	NT	NT	NT	—	100
	Inhibition zone (D)	NA	NA	NA	NA	NA	NA	NA	NA	NA	NA		
	Inhibition (%)	NA	NA	NA	NA	NA	NA	NA	NA	NA	NA		

Fluconazole^1^ (1 mg/mL)	MIC/MFC	2/64	32/128	16–32/ 32–64	16/64	8–16/ 62–128	32/32	64/128	64/>128	>128/>128	>128/>128	1.00	86 ± 3.6
	Inhibition zone (D)	16 ± 2.1	12 ± 1.2	15 ± 1.33	17 ± 1.14	16 ± 2.33	15 ± 2.67	15 ± 1.75	14 ± 2.6	2 ± 1.46	3 ± 1.85		
	Inhibition (%)	38.09 ± 2.1	30.77 ± 1.2	37.5 ± 1.33	53.13 ± 1.14	57.14 ± 2.33	55.56 ± 2.67	53.57 ± 1.75	60.87 ± 2.6	4.44 ± 1.46	7.5 ± 1.85		

*C*. *al: Candida albicans, S*. *ce: Saccharomyces cerevisiae, T*. *men: Trichophyton mentagrophytes, T*. *rub: Trichophyton rubrum, T*. *tons: Trichophyton tonsurans, E*. *flo: Epidermophyton floccosum, M*. *can: Microsporum canis, M*. *gyp: Microsporum gypseum, A*. *ni: Aspergillus niger, and P.* spp*: Penicillium* spp.

^1^Standard.

NT: none tested.

NA: not available.

Data are expressed as means ± standard deviation; *n* = 3.
